# Leveraging network analytics to infer patient syndrome and identify causal genes in rare disease cases

**DOI:** 10.1186/s12864-017-3910-4

**Published:** 2017-08-11

**Authors:** Andreas Krämer, Sohela Shah, Robert Anthony Rebres, Susan Tang, Daniel Rene Richards

**Affiliations:** QIAGEN Bioinformatics, 1001 Marshall Street, Suite 200, Redwood City, CA 94063 USA

**Keywords:** NGS, Whole-genome sequencing, Exome sequencing, Rare disease diagnosis, Variant selection, Genetic disorders, Diagnostic odyssey

## Abstract

**Background:**

Next-generation sequencing is widely used to identify disease-causing variants in patients with rare genetic disorders. Identifying those variants from whole-genome or exome data can be both scientifically challenging and time consuming. A significant amount of time is spent on variant annotation, and interpretation. Fully or partly automated solutions are therefore needed to streamline and scale this process.

**Results:**

We describe Phenotype Driven Ranking (PDR), an algorithm integrated into Ingenuity Variant Analysis, that uses observed patient phenotypes to prioritize diseases and genes in order to expedite causal-variant discovery. Our method is based on a network of phenotype-disease-gene relationships derived from the QIAGEN Knowledge Base, which allows for efficient computational association of phenotypes to implicated diseases, and also enables scoring and ranking.

**Conclusions:**

We have demonstrated the utility and performance of PDR by applying it to a number of clinical rare-disease cases, where the true causal gene was known beforehand. It is also shown that PDR compares favorably to a representative alternative tool.

**Electronic supplementary material:**

The online version of this article (doi:10.1186/s12864-017-3910-4) contains supplementary material, which is available to authorized users.

## Background

Whole genome and exome sequencing is widely used to identify disease-causing variants in patients with multiple congenital abnormalities and rare genetic disorders. However, a key challenge in using this approach is finding the true causal variants among the hundreds of rare, functional (coding and/or regulatory) variants. It can take many hours to evaluate the relationship between variants in a patient’s sequence data and his phenotype or disease, in order to identify the disease-causing mutation [1]. In addition, the disease-causing variant is successfully identified in only 25–30% cases [2, 3].

Here, we describe Phenotype Driven Ranking (PDR), an algorithm integrated into Ingenuity Variant Analysis (QIAGEN Bioinformatics, Redwood City, CA) [4] that uses observed patient phenotypes to prioritize diseases and genes in order to expedite causal-variant discovery. Our method is based on a network of phenotype-phenotype, phenotype-disease, and disease-gene relationships constructed from the QIAGEN Knowledge Base (KB) [5], and aims to identify diseases that can explain both the phenotypes observed as well as the genetic variations detected. The approach explicitly allows for traversal of a phenotype/disease hierarchy, which connects more specific phenotypes to more general ones, and thereby expands the search space of phenotype terms that can be associated with a given disease. For each disease, a score is computed that reflects the similarity between the phenotypic profile and disease, and this score is in turn used to rank variants that reside in disease-implicated genes.

A number of tools, such as Phenolyzer [6], Phevor2 [7], Phen-Gen [8], GeneCards [9], and Exomiser [10] leverage databases of gene-disease-phenotype relationships and phenotype information to prioritize candidate genes. All of these tools, including PDR, require a list of phenotypes as input, either in the form of HPO identifiers [11] or clinical terms, to generate a ranked list of genes based on the plausibility of being associated with those phenotypes. Using next-generation sequencing data (e.g. supplied as a VCF file), this gene list can then be further trimmed down by focusing on relevant variants. Some tools leverage integration with other tools to provide this capability (Phevor2 + VAAST [12], Phenolyzer + ANNOVAR [13], PDR + Ingenuity Variant Analysis), while others offer it as part of the tool (Phen-Gen, Exomiser). Similarly, by partnering or independently, many of these tools (Phevor2 + VAAST, PDR + Ingenuity Variant Analysis, Phen-Gen, Exomiser) can provide family-based analysis involving several samples, useful for identifying variants compatible with the disease inheritance mode. With the exception of Exomiser, all of the tools mentioned are web-based with content updates to reflect new gene-disease and disease-phenotype relationships in the computations.

A few studies [6, 9] have benchmarked some of these existing tools against each other. To put our method in context, in this paper we will carry out a comparison of PDR and Phenolyzer by assessing the detection of previously known causal genes in rare disease cases. Phenolyzer, in contrast to Phen-Gen and Exomiser, operates on the gene level (not variant-level) and therefore uses the same input as the PDR algorithm, making it best suited for this comparison. Moreover, Phenolyzer compared favorably to Phevor2 and GeneCards for monogenic diseases [6], which suggests it as ideal benchmark for PDR’s targeted use case.

## Methods

### Ingenuity variant analysis

Ingenuity Variant Analysis is a web-based application to annotate and filter whole-genome and exome sequencing data using variant quality metrics (call quality, read depth, genotype quality, etc), population allele frequencies (using 1000 Genomes [14], NHLBI-EVS [15], ExAC [16], Allele Frequency Community^1^ [17]), known pathogenicity (published literature and HGMD [18]), variant type (coding, regulatory, non-coding, loss or gain of function, etc), inheritance models, gene-disease relationships, gene functions, and pathways. As a general concept, data is piped through a cascade of several filters, each letting pass only a subset of variants (and associated genes) that fulfill certain criteria. Examples are filters for common variants, variant call confidence, inheritance models, functional impact, statistical analysis, and filters relating to prior biological information.

All analyses reported here used the following pre-configured settings of the filter cascade. Variants are filtered to remove low quality calls (call quality <20) and common variants (>0.5% MAF in 1000 Genomes, NHLBI-EVS, ExAC, and Allele Frequency Community). We keep only variants that have previously been published as pathogenic or likely pathogenic using ACMG guidelines [19], are DM variants (i.e. pathological mutations reported to be disease causing in the original literature report), from HGMD, are associated with a loss or gain of function (frameshift, start/stop loss or gain, splice site), or are missense variants.

### Phenotype-disease-gene network

The PDR algorithm is based on a large-scale network (directed acyclic graph) whose nodes consist of diseases, phenotypes, and genes (see Additional file 1: Table S1). These nodes are connected by three types of directed edges, gene-disease (GD) edges, disease-phenotype (DP) edges, and process hierarchy (PH) edges corresponding to underlying content and ontology structure of the QIAGEN KB. GD edges connect genes with associated diseases (either causal or correlated) and are based on literature-curated findings, databases (OMIM [20], GO [21], MGI [22], ClinVar [23], HGMD, HMDB [24], GVK [25], COSMIC [26]), curated information about clinical trials, and drug labels. DP edges link diseases to associated phenotypes and reflect content from HPO and OMIM, and to a lesser extend QIAGEN-internal assertions and curation from the literature. Finally, PH edges connect more general to more specific terms in the functional annotation hierarchy of the QIAGEN KB depending on internal modeling but referencing many external sources (e.g. NCI [27], SNOMED [28], FMA [29] and others). Note that there is no strict distinction between diseases and phenotypes since some phenotypes act as diseases themselves, i.e. can be connected to other downstream phenotypes through DP edges. Overall, the network^2^ contains 4811 diseases (connected to at least one phenotype), 5843 phenotypes (connected to at least one disease, 348 of which are diseases themselves), and 18,070 genes (associated with at least one disease). Each gene is on the average associated with 6.8 diseases (maximal 157). Diseases have on the average 43.4 associated phenotypes (maximal 455), while phenotypes are on the average associated with 35.9 diseases (maximal 1403). The process hierarchy contains 106,223 biological functions including phenotypes, connected by 208,933 (PH) edges. There are 190,993 DP edges, and 122,538 GD edges in the network.

### Mapping of phenotypes

Users enter a phenotype term as free-text or provide an HPO identifier in standard format (e.g. HP:0000213). As a term is entered, the QIAGEN KB supplies phenotypes matching the text as an autocompleted entry or as alternatives for selection. Spacing, capitalization, and hyphenation are normalized during fuzzy-matching of entered terms. Supported phenotype terms include all names and synonyms for any disease, abnormality, or biological process computationally associated with findings in the QIAGEN KB. More than 60,000 phenotypes are available, including 44,000 phenotypes associated with variants in the QIAGEN KB. More than a half-million unique phenotype synonyms are derived from a variety of sources including Snomed, NCI, Orphanet [30], MeSH [31], ICD [32], HPO, MPO [33], GO, and the literature. Phenotypes mapping to HPO concepts are integrated into the larger QIAGEN KB phenotype hierarchy. HPO phenotypes cited in 92% of the phenotype annotations described by HPO or Orphanet for OMIM or Orphanet diseases are currently supported. For supported HPO phenotypes, both primary and alternate identifiers, as well as primary term and all synonyms, are available for mapping. Support for HPO terms has been prioritized based on frequency of their use in phenotype annotations, and improvements in coverage are ongoing.

### PDR workflow

**Fig. 1:**
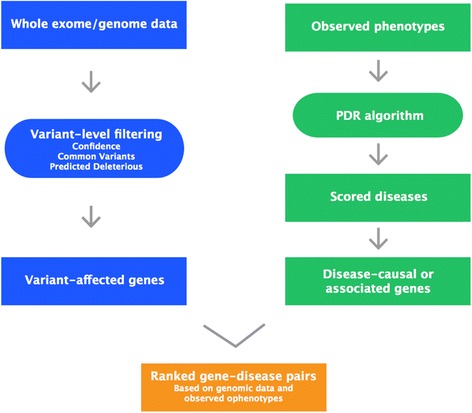
Workflow schematic of Phenotype-Driven Ranking. PDR uses both, genomic data and observed phenotypes to infer likely diseases and their associated causal or correlated variant-affected genes. Only those genes are considered that pass Confidence (filtering by call quality, read depth, and genotype quality), Common Variant (filtering by population allele frequencies using 1000 Genomes, NHLBI-EVS, ExAC, and Allele Frequency Community), and Predicted Deleterious (filtering by known pathogenicity from published literature and HGMD, and variant type like coding, regulatory, non-coding, or loss or gain of function) filters in Ingenuity Variant Analysis. PDR itself employs a heuristic scoring algorithm that is based on linking phenotypes to diseases in a large-scale, hierarchical network of phenotype-phenotype, and phenotype-disease relationships

The overall workflow of the PDR algorithm is shown in Fig. 1. Whole-genome or exome data is analyzed in Ingenuity Variant Analysis and results in a set of variant-impacted genes depending on the settings of the variant-filter cascade. Observed phenotypes are fed into the PDR algorithm and mapped to a scored list of diseases defining a set of associated causal or correlated disease genes. Both gene sets, variant-impacted genes and disease genes, are then intersected to result in a final set of genes and their associated scored diseases. In the practical implementation, for computational efficiency, the PDR algorithm probes only the subset of diseases with variant-impacted disease genes, which is equivalent to the set intersection described above.

### Network algorithm and scoring heuristic

For each disease, the PDR algorithm computes a heuristic score *S* aimed at measuring disease relevance in the context of the observed phenotypes. This score is defined as a weighted count of phenotypes that can be connected to the disease through the phenotype-disease network. Phenotype-associated weights take into account two contributions: (1) the prevalence among all diseases represented in the QIAGEN KB (called “specificity weight”), and (2) the confidence of relating a phenotype to a disease when traversing the process hierarchy (called “path weight”). The specificity weight ${w}_s^i$ for a phenotype *i* is given by

1 $$ {w}_s^i=\frac{1}{1+ b\ { \log}_{10}\left( \max \left(1,{N}_i\right)\right)} $$

where *N*_*i*_ is the number of diseases that the phenotype *i* is directly connected to in the network, and the parameter *b* is set to 1. The value of the specificity weight is 1 for a phenotype that is directly connected to either 0 or 1 diseases, and becomes smaller if the phenotype is less disease-specific, i.e. the number of connected diseases increases. The distribution of specificity weights for all phenotypes is shown in Additional file 1: Figure S2.

For a given disease, the path weight ${w}_p^i$ of a phenotype *i* is calculated as

2 $$ {w}_p^i={a}^{L_i-1} $$

with *L*_*i*_ being the length of the shortest path from a phenotype *i* to the disease node in the phenotype-disease network, and *a* is set to 0.75. For direct phenotype-disease links, and in the special case where a phenotype is a gene-associated disease itself, the path weight is set to 1. For longer paths that weight decreases. The maximal considered path length when traversing the phenotype-disease hierarchy is 4. The score *S* for a given disease is then computed as the sum over all connected phenotypes *i*,

3 $$ S=\sum_i{w}_s^i{w}_p^i $$

Given a set of genes *G*, and a set of phenotypes *P* as input, the PDR algorithm consists of the following steps:

1. Determine the set *D* of diseases correlated with or caused by genes in *G*.
2. From any given phenotype in *P*, determine all shortest paths to a disease in *D* under the condition that the path does not contain other diseases in *D* unless it is identical to the given phenotype, and the last edge in the path is a phenotype-disease relationship.
3. For every disease *d* in D, collect all paths from step 2. connecting *d* to phenotypes in *P*, compute the score *S* defined above, and also combine all shortest paths into a sub-network for visualization. Diseases in *D* that cannot be connected to any phenotype in *P* (within a maximal path length of 4) are dropped.

The result of this algorithm is a list of diseases (a subset of *D*) with their associated score *S*, and one or more correlated or causally associated genes from *G*.

Our motivation for the heuristic algorithm and score described above is the following: By summing over user-supplied phenotypes connected to a given disease, the score *S* essentially measures the amount of “evidence” we have that the disease is in fact a cause for the observed phenotypes; the higher the score *S*, the more phenotypes can be “explained”. However, more specific phenotypes are weighted higher in this sum, since they more likely discriminate between competing diseases, while phenotypes that can be connected only through longer paths are weighted lower, since our confidence of disease-phenotype association decreases with each link traversed. The use of a logarithm in Eq. (1) for the specificity weight stems from the intuition, that the value of this weight should roughly reflect the order of magnitude of the number of phenotype-associated diseases, i. e. measure the qualitative difference between phenotypes associated with – say – 1, 10, 100, or 1000 diseases, without suppressing unspecific phenotypes entirely. The two parameters *a*, and *b* in Eq. (1) and (2) determine how fast specificity and path weights decrease with the number of connected diseases and path length respectively. We have tested a range of values for *a,* and *b* (set to *a* = 0.75 and *b* = 1 in the final implementation) in a number of practical use cases and found results to be fairly robust w.r.t. parameter choice, except in extreme cases (for instance setting *a* close to 1 would bring up more diseases that are only loosely associated with the supplied phenotypes as high-scoring). It shall be noted, that the score *S* cannot distinguish situations where a disease is connected to many unspecific phenotypes, from cases where the disease is connected to a few specific ones, with the trade-off between the two depending on the actual choice of the parameters *a* and *b*. Performance of the PDR algorithm crucially depends on the quality and extent of the underlying phenotype-gene-disease network. While the process hierarchy allows to extend disease-phenotype relationships beyond those that were explicitly curated from the literature, the network contains only well-established disease-gene relationships; therefore the algorithm cannot predict novel disease genes.

### User-interface, result table, and network visualization

**Fig. 2:**
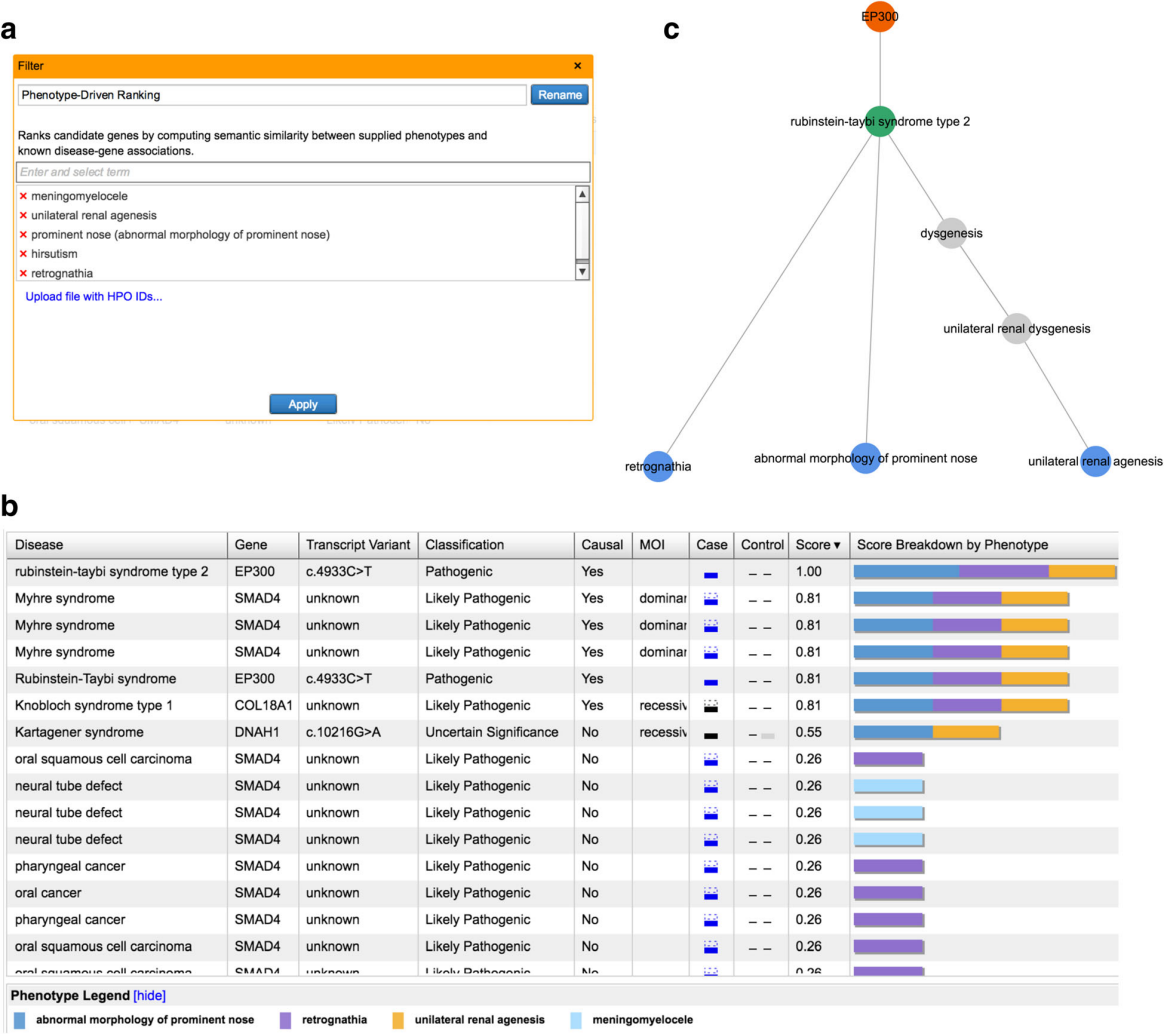
User interface of PDR in Ingenuity Variant Analysis. **a** Users enter phenotypes through a widget that employs autocomplete functionality as well as synonym mapping, and also recognizes HPO identifiers. **b** Results after running the PDR algorithm are displayed in a table that is rank-ordered by disease score, where rows correspond to unique disease-gene-variant combinations. Each row displays the following information (table headers shown in parentheses): inferred disease (“Disease”), associated gene (“Gene”), variant information if available (“Transcript Variant”), variant pathogenicity classification according to ACMG guidelines (“Classification”), the nature of the gene-disease relationship from OMIM (“Causal”), mode of inheritance (“MOI”), a graphical representation of variant zygosity and functional impact if known (“Case”), and the disease score from Eq. (3) (“Score”), as well as a visual representation of its contributing components by phenotype (“Score Breakdown by Phenotype”). Note, that the picture shown here is an actual screenshot of the result table as it is displayed in Ingenuity Variant Analysis, which also contains a column labeled “Control”. This column has no meaning in the context of the present paper. **c** Selecting a table row in the user interface leads to the display of the corresponding gene-disease-phenotype sub-network that, besides the causal or disease-correlated gene (*orange*), the inferred disease (*green*), and the linked phenotypes (*blue*), may also contain intermediate nodes of the phenotype-disease hierarchy (*gray*)

PDR is integrated into the filter cascade in Ingenuity Variant Analysis and takes as input the list of genes (and associated variants) that pass the preceding filters. When setting up the PDR filter, users can enter observed phenotypes (including HPO terms) into a widget that performs term mapping through an autocomplete function (an example is shown in Fig. 2a). After running an analysis, results are displayed in a table (Fig. 2b) where diseases are rank-ordered by score. Each table row contains a disease with associated causal or correlated gene and variant. If a disease has several corresponding genes, or a gene has several (filter-passing) variants (maximal 5 shown), table rows are simply replicated. The table is truncated at the 50 highest-scoring disease/gene pairs.

Each table row displays a visual representation of the score indicating the individual phenotype contributions. Also shown is information about the nature of the gene-disease relationship (causal or not causal, derived from OMIM and other sources), as well as the mechanism of inheritance if known, along with variant properties (ACMG classification, zygosity, inferred loss or gain of function). Note, that the numerical value of the score only depends on the disease and its supporting phenotypes. We have chosen not to integrate gene or variant properties into the score itself, but rather allow the user to evaluate phenotypic evidence (expressed by the score), and gene- or variant-level evidence independently.

When selecting a table row in the application’s user-interface, a visualization of the supporting sub-network is shown, displaying all shortest paths from phenotypes to corresponding disease and gene through the phenotype-disease-gene network (see Fig. 2c). For context and supplementary evidence, this network can also display additional edges linking gene and (possibly) intermediate phenotypes directly if that information is available in the QIAGEN KB. Note, that these additional edges are not used in the PDR algorithm itself.

The computational performance of the PDR algorithm is of the order of seconds on a typical server.

## Results and discussion

We have tested and benchmarked PDR with 27 patient cases from Inova Translational Medicine Institute, Fairfax, VA (ITMI). Some of these cases have been studied and published elsewhere in different contexts [34–40]. For the present analysis, fastq files were used to call variants within the BxWB hereditary disease pipeline [41] that directly exports data to Ingenuity Variant Analysis. Here, we set up a filtering and interpretation pipeline as described in the Methods section using best practices guidelines. For all 27 cases, the causal variant and gene had previously been determined with very high confidence by the ITMI clinical genetics team based on manual review of relevant literature, and additional information not used by PDR itself, like inheritance and occurrence of structural variants such as de novo large deletions. Additional file 2: Table S3 lists physician-reported phenotypes as well as reported causal disease genes for all 27 cases.

**Fig. 3:**
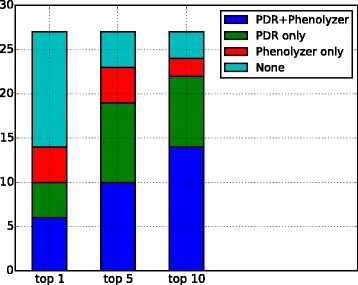
Ranking of previously known causal genes: comparison of PDR and Phenolyzer [6, 42]. The stacked bar chart shows the number of cases (among the 27 cases studied) in which the known causal gene could be recovered among the top 1, top 5, and top 10 ranking genes

As an example, results for one of the analyzed cases, which has also been published previously [35], is shown in Fig. 2a-c. In this case, five observed phenotypes, meningomyelocele, unilateral renal agenesis, prominent nose, hirsutism, and retrognathia, were entered into PDR (Fig. 2a). The workflow included the filter cascade described in the Methods section. In addition, we only looked at de novo variants in the probands, as whole genome sequence data for both parents was also available. The known causal gene and diagnosed disease, EP300 and Rubinstein-Taybi Syndrome, are found at the top position in the rank-ordered table (Fig. 2b). When all genes with rare, deleterious variants in the proband are passed to the PDR filter without applying the inheritance model, EP300 ranks fourth. Figure 3c shows the corresponding phenotype-disease-gene subnetwork. Note, that one of the user-supplied phenotypes is not directly connected to the disease, but through two intermediate terms (dysgenesis, and unilateral renal dysgenesis) in the disease/phenotype hierarchy.

### Detection and ranking of known causal disease variants and genes using PDR

In order to benchmark our approach, we examined whether known causal genes could be recovered by PDR alone using the provided patient phenotypes in each of the 27 cases (see Additional file: 2 Table S3). In 22 out of 27 cases, the reported causal gene ranked within the top 10 genes inferred by PDR. In 9 cases, the previously reported gene was also the highest scoring one, and variants present in the causal gene were consistent with the disease mode of inheritance (heterozygous for dominant genes and homozygous or compound heterozygous for recessive). In the remaining cases, the causal variant could be identified using a combination of the disease score, causal relation between the genes and diseases, consistency between variant genotype and mode of inheritance, and the computed ACMG classification. In 4 out of 27 cases, PDR could not identify the correct gene.

It is interesting to ask, why in some cases the disease associated with the known causal gene scored lower than other diseases. Closer examination revealed, that in about half of those cases, other diseases could be explained by a greater number of supplied phenotypes, while in the other half the number of explaining phenotypes was the same, but some of them were more specific or could be connected to the disease through shorter paths. This underscores the sensitivity of the disease score w.r.t. the proper selection of input phenotypes. Providing a more complete set of observed phenotypes, or supplying more specific ones, will more likely discriminate the actual disease (and its causal gene) from others. In practice, it is usually sufficient that the actual causal gene, variant, and disease scores near the top of the list (not necessarily in first place) since information other than phenotypes, for example mode of inheritance, zygosity, and variant classification, can be used to distinguish it from other high-scoring disease/gene/variant combinations.

We want to point out that for two of the 27 patient test cases considered here the corresponding publications, [35, 37], were previously curated into the QIAGEN KB, and support causal gene-disease relationships EP300 ➔ Rubinstein-Taybi syndrome, and NOTCH1 ➔ Adams-Oliver syndrome in the network. However, this does not introduce data circularity, since those two relationships are also each supported by more than 10 other independent literature findings. Findings from [35] and [37] could therefore simply be removed from the KB with no effect on our results.

### Comparison analysis

Here, we compare PDR to Phenolyzer, a widely used tool to determine likely causal genes from observed phenotypes, which has also been shown to perform well compared to other, similar tools [7] (see Background section). In addition to phenotypes, Phenolyzer can also be provided with a set of target genes which will then be the only ones scored. To carry out the comparison analysis, for each of the 27 patient cases described above, we determined the set of genes (including the known causal gene) that was used as input into the PDR algorithm within Ingenuity Variant Analysis (i.e. those genes that pass the preceding filter cascade), and used those genes as input into the Phenolyzer web interface [42] with default parameter settings.

When directly entering phenotype terms from Additional file 2: Table S3 into Phenolyzer, on the average only 62% of them could be recognized directly. We therefore tried to map all provided phenotypes to corresponding HPO identifiers first in a pre-processing step (using also synonym mapping), before entering them into Phenolyzer, which on the average was successful for 92% of the input terms. For each of the 27 patient cases, we then determined the rank of the previously known causal gene, when all genes are ranked by score, for both PDR and Phenolyzer (for details see Additional file 2: Table S3). Figure 3 shows the result of the comparison analysis for causal genes ranked as top 1, top 5, and top 10. It is seen that, when only looking at top 1-ranking genes, about half of the causal genes are missed by both, PDR and Phenolyzer, with the remaining genes found either by both, PDR only, or Phenolyzer only in about equal proportions. For top 10-ranking genes, both PDR and Phenolyzer find the majority of causal genes, however, for causal genes only discovered by one of the tools, PDR is seen to have an advantage. In total, both tools recover 10 out of 27 genes as top-ranking, while PDR finds the causal gene in 22 out of 27 cases among the top 10, compared to 16 out of 27 for Phenolyzer.

When interpreting these results, it needs to be noted that the algorithms underlying both tools, while similar in mapping phenotypes to diseases and then to causal genes, differ in key aspects. PDR likely has the substantial advantage of relying on a powerful ontology and being able to “propagate” phenotype terms through a disease/phenotype hierarchy to find impacted diseases. Phenolyzer, on the other hand, expands its search space for disease-associated genes beyond known disease-gene associations from the literature (as PDR does) by employing gene-gene relationships like protein-protein binding. It is possible that we will implement a similar feature for PDR in the future.

## Conclusions

The Phenotype-Driven Ranking filter (PDR) in Ingenuity Variant Analysis uses phenotypes to infer and rank matching diseases and enables prioritization of disease-causing variants and genes from whole genome and exome sequence data for individuals with genetic disorders. Apart from variant and gene prioritization, PDR can also be used as a diagnostic aid that enables fast and accurate disease prediction based on clinical signs and symptoms observed alongside genotype information. We demonstrate here that PDR performs well for a number of clinical cases where the causal gene was known previously, and also show how it compares to a representative existing tool.

## Additional files

Additional file 1: Supplementary Information. **Table S1.** Disease-related content metrics of the QIAGEN KB; **Figure S2.** Phenotype specificity weight distribution; and a description of column headers used in Additional file 2: Table S3. (DOCX 143 kb)

Additional file 2: Table S3. This table lists the detailed results used for benchmarking and comparison analysis. In particular, for all 27 patient cases it shows the observed clinical phenotypes used as input, as well as all corresponding previously known causal genes. It also shows ranking of causal genes in PDR (after running analyses in Ingenuity Variant Analysis taking whole genome data as input), the corresponding disease inferred by PDR, and causal variant properties. For comparison, the rank of the causal gene obtained from Phenolyzer is also shown. (XLSX 34 kb)

## Footnotes

1 The Allele Frequency Community [17] is a hosted allele frequency database, founded by QIAGEN and participating members in 2014. It is a freely accessible “opt-in” community resource designed to facilitate sharing of anonymized, pooled allele frequency statistics among community members.

2 Numbers below represent a snapshot at the time of manuscript submission, and will likely change due to content updates of the QIAGEN KB in the future.
