# Supplementary material for: Leveraging network analytics to infer patient syndrome and identify causal genes in rare disease cases
Source: BMC Genomics. 2017 Aug 11;18(Suppl 5):551. doi: 10.1186/s12864-017-3910-4 (PMC5558185; doi:10.1186/s12864-017-3910-4)
Supplement: Supplementary file 1 — Supplementary Information. Table S1. Disease-related content metrics of the QIAGEN KB; Figure S2. Phenotype specificity weight distribution; and a description of column headers used in Additional file 2: Table S3. (DOCX 143 kb) [file 12864_2017_3910_MOESM1_ESM.docx]

**Supplementary Information**

**Table S1**

**Disease-related content metrics of the QIAGEN KB (Nov 16, 2016)**

| gene-level findings relating to diseases and disorders | 1,028,287 |
| --- | --- |
| gene-scope homolog subtypes (including isoform-level) | 32,591 |
| human genes (roll-up of from isoforms) | 18,801 |
| murine genes (contributing to gene-scope homologs) | 6,953 |
| types of disease | 31,692 |
| types of disease (for human genes) | 21,583 |

**Figure S2**

**Phenotype specificity weight distribution**

Histograms below show the overall distribution of the number of phenotype-associated diseases (left) and the resulting distribution of phenotype specificity weights (right) for *b* = 1 (see Eq. 1).


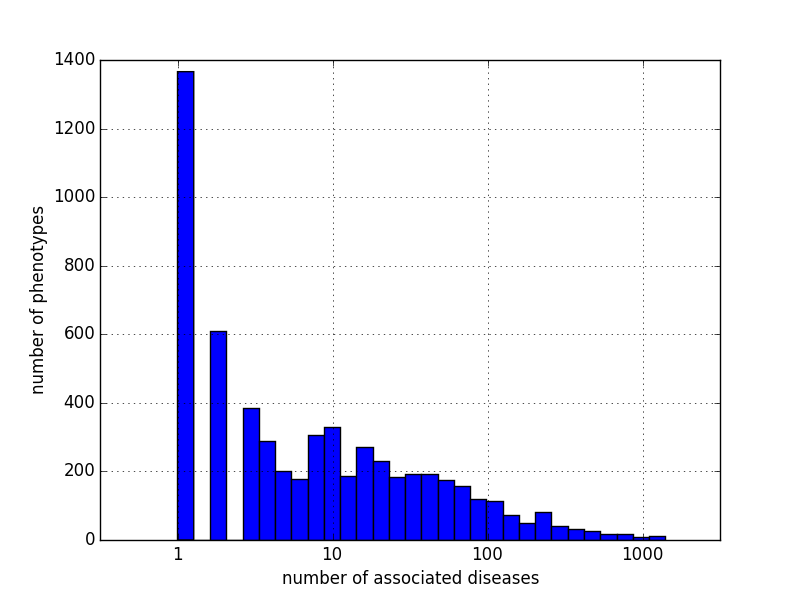

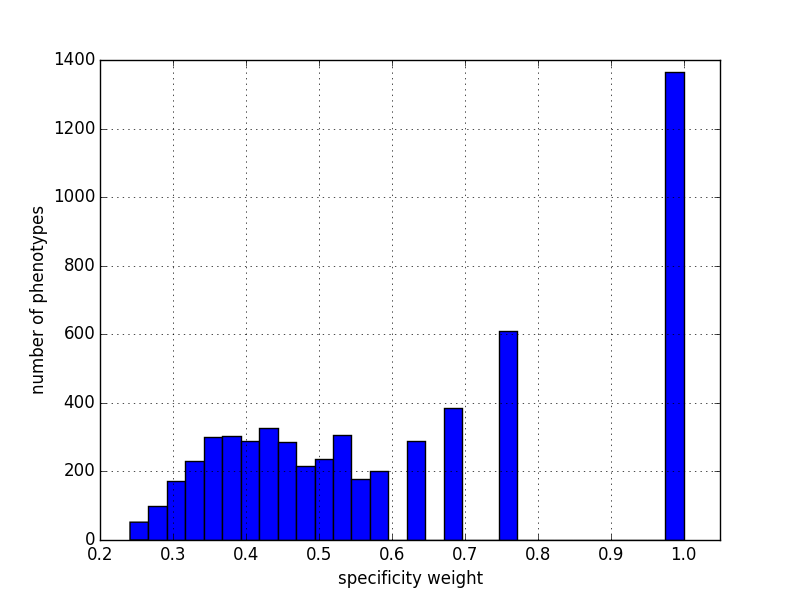


**Description of column headers used in Table S3**

*Input:*

1. **Case number**
2. **Observed phenotypes**
3. **Known causal gene**

*PDR output for known causal gene*

1. **Inferred disease**
2. **Is causal?** (yes/no)
3. **MOI** (mode of inheritance: dominant/recessive/X-linked)

*PDR benchmarking and comparison to Phenolyzer*

1. **PDR rank**

Ranking by score, but tie-breaking takes obvious variant and gene properties into account (e.g. homozygous, heterozygous/dominant, predicted pathogenic rank higher than heterozygous/recessive, predicted benign)

1. **PDR rank (gene information only)**

For comparison to Phenolyzer: ranking by score, but tie-breaking takes gene properties into account (causal ranks higher the non-causal, then dominant ranks higher than recessive ranks higher than X-linked)

1. **Phenolyzer rank**
2. **Number of genes passing preceding filter cascade in Ingenuity Variant Analysis**

*Properties of causal variant*

1. **Computed ACMG classification** (pathogenic/likely pathogenic/VUS/likely benign/benign)
2. **Zygocity** (homozygous/heterozygous)
3. **Predicted functional effect** (loss/gain)
